# Identification of the superficial peroneal nerve: Anatomical study with surgical implications

**DOI:** 10.1007/s00167-016-4063-8

**Published:** 2016-03-26

**Authors:** Peter A. J. de Leeuw, Pau Golanó, Leendert Blankevoort, Inger N. Sierevelt, C. Niek van Dijk

**Affiliations:** Department of Orthopaedic Surgery, Orthopaedic Research Center Amsterdam, Academic Medical Center, University of Amsterdam, PO Box 22700, 1100 DE Amsterdam, The Netherlands; Academic Center for Evidence based Sports medicine (ACES), Amsterdam, The Netherlands; Amsterdam Collaboration for Health and Safety in Sports (ACHSS), Amsterdam, The Netherlands; Laboratory of Arthroscopic and Surgical Anatomy, Department of Pathology and Experimental Therapeutics (Human Anatomy Unit), University of Barcelona, Barcelona, Spain; Orthopedic Surgery Department, Medicine School, University of Pittsburgh, Pittsburgh, PA USA; Orthopedic Department, MC Slotervaart, Louwesweg 6, 1066 EC Amsterdam, The Netherlands

**Keywords:** Superficial peroneal nerve, Anatomy, Ankle arthroscopy, Complications, Body mass index

## Abstract

**Purpose:**

To prevent iatrogenic damage to the superficial peroneal nerve during ankle arthroscopy, it needs to be identified. The purpose of the present study was to determine which clinical test identified the superficial peroneal nerve most frequently and which determinants negatively affected the identification.

**Methods:**

A total of 198 ankles (99 volunteers) were examined for identification of the superficial peroneal nerve. Race, gender, body mass index (BMI), shoe size and frequency of physical activity were collected.

**Results:**

The best method to identify the superficial peroneal nerve was the maximal combined ankle plantar flexion and inversion test. In this position, the nerve was identified in 57 % of the ankles by palpation. BMI was the only independently influential factor in the identification of the superficial peroneal nerve.

**Conclusion:**

Since in nearly six out of the ten ankles the superficial peroneal nerve can be identified, it is advised to assess its anatomy prior to portal placement. A higher BMI negatively influences the identification of the superficial peroneal nerve.

**Level of evidence:**

Diagnostic study, Level III.

## Introduction

Anterior ankle arthroscopy is routinely used as a minimally invasive surgical technique for the treatment of ankle pathology. Sine introduction, arthroscopic equipment has improved significantly, enabling surgeons to visualize and treat an increasing number and more complex pathologies [25, 26].

Complication rates for anterior ankle arthroscopy have been reported to be up to 17 %, of which more than 25 % was related to iatrogenic superficial peroneal nerve damage during anterolateral portal placement [2, 4, 6, 8, 10–13, 14, 16–18, 21, 27]. The superficial peroneal nerve perforates the crural fascia at an average of 13 cm proximal to the level of the ankle joint [1]. Subsequently, the nerve divides into its terminal branches: the medial dorsal cutaneous and the intermediate dorsal cutaneous nerves [14, 17]. The level at which this nerve penetrates the fascia and the level at which the terminal branches originate has been studied extensively [1, 5, 15, 20, 24].

The superficial peroneal nerve and the above-mentioned branches are the only nerves in the human body that can be visualized at clinical examination [22]. Identification of the superficial peroneal nerve preoperatively may prevent iatrogenic damage [23]. Literature is inconclusive with respect to which specific test identifies the superficial peroneal nerve most accurately. Furthermore, it is unknown which determinants influence nerve identification. Determinants which have a negative influence on the identification of the superficial peroneal nerve might account for an increased risk of superficial peroneal nerve damage following surgery.

The purpose of this study was to determine which specific clinical tests identify the superficial peroneal nerve most frequently and which determinants negatively affected its identification. We hypothesized that in the majority of the population the superficial peroneal nerve, or if divided, its terminating branches, can be identified by ankle plantar flexion and inversion.

## Materials and methods

Inclusion criteria were volunteers between 18 and 40 years old, with the ability to understand and sign the informed consent form. Informed consent was obtained in all volunteers. Volunteers were excluded if they had a history of foot and/or ankle surgery.

The investigator was trained on cadaveric specimens in the specific clinical examinations by an experienced anatomist (PG). The superficial peroneal nerve was distinguished from blood vessels by its subcutaneous colour and the fact that vessels, in contrast to nerves, can be compressed. Arteries are not located subcutaneously; thus, only veins were relevant in this examination. The subcutaneous colour of the nerve is white, while veins are coloured blue in the Caucasian and Asian people. Distinguishing the superficial peroneal nerve from the extensor tendons was done by flexion and extension of the toes.

**Fig. 1:**
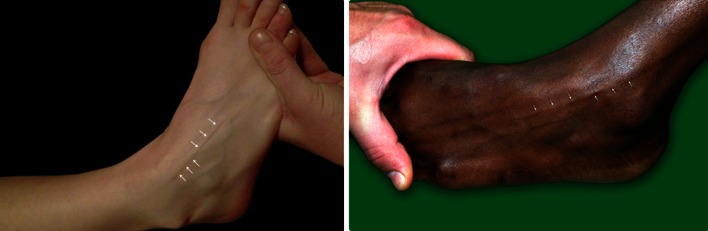
Visible identification of the superficial peroneal nerve (*white arrows*) by combined ankle plantar flexion and inversion both in an ankle from the Caucasian and Negroid race

Superficial peroneal nerve visibility and palpability were assessed in different foot and ankle positions: the neutral (90°) position, same ankle position but with the addition of maximal plantar flexion of the fourth toe, and the forced combined maximal ankle plantar flexion and inversion position (Fig. 1).

Race, gender, and physical activity of the volunteer were determined. The height and weight of each volunteer were recorded, and the BMI was subsequently calculated. The European shoe sizes and the frequency of sport participation were recorded following a questionnaire. Institutional review board approval from the University of Amsterdam, the Netherlands, was obtained under registration number 08/053.

### Statistical analysis

Statistical analysis was performed using SPSS 19.0 (SPSS Inc., Chicago, Illinois, USA). Identification of the peroneal nerve in three positions (neutral, fourth toe flexion and combined plantar flexion/inversion) is presented as proportions (with 95 % CI) and pairwise tested using McNemar tests to determine which clinical test is most sensitive to identify the superficial peroneal nerve. Additionally, a post hoc power analysis was performed.

A mixed logistic regression analysis was performed to assess the influence of the determinants (gender, age, race, shoe size, and BMI) on the detectability of the superficial peroneal nerve. To account for dependency between left and right leg within the individual, the variance–covariance structure “compound symmetry” was used. Odds ratios (with 95 % CI) were calculated for the factors that were significantly (*p* < 0.05) associated with the identification of the superficial peroneal nerve.

## Results

**Fig. 2:**
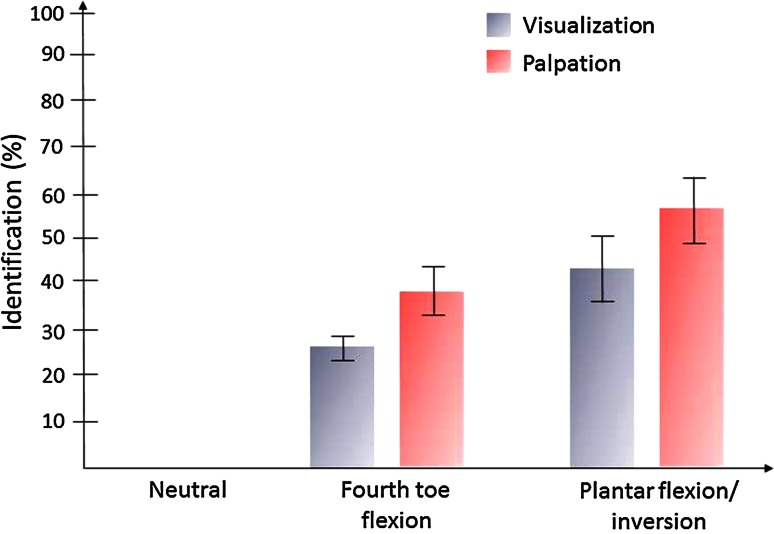
Percentage of the ankles in which the superficial peroneal nerve could be identified (with 95 % CI) for the foot in the neutral position and for each of the clinical tests

We recruited 99 volunteers (39 men, 60 women), in whom both ankles were examined (*n* = 198). On examination with the ankle in the 90° position, the superficial peroneal nerve was neither visible nor palpable in any of the ankles. With the addition of fourth toe flexion, the superficial peroneal nerve, or its terminal branches, was identified by visualization in 27 % (95 % CI 21–33 %) of the ankles (*n* = 54) and was palpable in 38 % (95 % CI 33–43 %) of the ankles (*n* = 75). With the ankle in the combined maximal plantar flexed and inverted position, the visibility and palpability of the superficial peroneal nerve were, respectively, 41 % (95 % CI 34–48 %) (*N* = 81) and 57 % (95 % CI 50–64 %) (*n* = 113) (Fig. 2). Palpation in the combined plantar flexion/inversion position was the most sensitive method to assess the location of the superficial peroneal nerve (*p* < 0.001); visualization of the nerve was only possible in the ankles in which the nerve was also palpable. In the ankles in which the nerve was identified with fourth toe flexion, also the maximal plantar flexion and inversion the test was positive. Post hoc power analysis revealed a power of more than 99 % to detect the differences between the three positions (90° position, fourth toe flexion, plantar flexed, and inverted position) for both identification procedures (visualization and palpation).

BMI was the only significant determinant of visibility and palpability of the superficial peroneal nerve (*p* = 0.001 and 0.04), respectively, Table 1) with odds ratios of 0.68 (95 % CI 0.55–0.85) and 0.83 (95 % CI 0.69–0.99), respectively. This implies that for each unit the BMI increases and the odds of identification of the superficial peroneal nerve decreases with 32 and 17 %, respectively.

**Table 1:** Determinants for identification by palpation of the superficial peroneal nerve in the maximal combined plantar flexion and inversion position following the mixed logistic regression analysis

	Volunteers (*n* = 99) (198 ankles)	Peroneal nerve identified (*n* = 113)	Peroneal nerve not identified (*n* = 85)	*p* value
*Gender*				
Male	39 (39 %)	44 (39 %)	34 (40 %)	0.96 (ns)
Female	60 (61 %)	69 (61 %)	51 (60 %)	
Age (mean, SD)	27.5 (5.9)	27.4 (6.1)	27.5 (5.7)	0.88 (ns)
*Race*				
Asian	33 (33.3 %)	45 (40 %)	21 (25 %)	.^a^
Negroid	33 (33.3 %)	30 (26 %)	36 (42 %)	0.05 (ns)
Caucasian	33 (33.3 %)	38 (34 %)	28 (33 %)	0.34 (ns)
Shoe size (mean, SD)	39.9 (2.6)	39.6 (2.5)	40.3 (2.7)	0.23 (ns)
*Sports participation*				
None	32 (32.3 %)	35 (31 %)	29 (34 %)	.^a^
Once a week	15 (15.2 %)	17 (15 %)	13 (15 %)	0.70 (ns)
Twice a week	27 (27.3 %)	27 (24 %)	26 (31 %)	0.67 (ns)
More	25 (25.3 %)	34 (30 %)	15 (18 %)	0.46 (ns)
BMI (mean, SD)	22.5 (3.8)	21.8 (2.0)	23.4 (5.2)	0.04

*ns* non-significant

.^a^Reference category

In the ankles in which the superficial peroneal nerve was visible, the mean BMI was 21.3 (SD 1.8) and the nerve was palpable in ankles with a mean BMI of 21.8 (SD 2.0). If the superficial peroneal nerve was not visible or palpable, the mean BMI was 23.3 (SD 4.5) and 23.4 (SD 5.2), respectively.

## Discussion

The most important finding of the present study was that the identification of the superficial peroneal nerve should ideally be performed in ankle plantar flexion and inversion, revealing this nerve in 57 % of the cases. The nerve was more frequently identified by palpation than by visualization. The fourth toe flexion sign did not aid in revealing a nerve that had already been identified with combined maximal plantar flexion and inversion. BMI was the only statistically significant determinant influencing identification of the superficial peroneal nerve.

According to Stephens and Kelly [22], the superficial peroneal nerve can be identified with the fourth toe flexion sign in 85 % of the volunteers. In our study, we could not reproduce this figure. Once they had clinically identified the nerve, an infiltration with a local anaesthetic fluid followed. Identification was deemed positive if subsequent skin numbness was experienced in the superficial peroneal nerve supply area. It is, however, questionable whether such an infiltration is accurate enough in precise identification of the nerve. The anaesthetic agent at the infiltration site could diffuse widely, resulting in nerve inhibition and thereby a positive fourth toe flexion sign. Local anaesthetic diffusion could in our view result in false-positive values, overestimating the test results. Furthermore, it was suggested that in women the superficial peroneal nerve is more difficult to identify because of their increased amount of subcutaneous fat [22]. Gender was not a significant determining factor for the identification of the superficial peroneal nerve in our study. However, the amount of subcutaneous fat at the anterolateral part of the ankle joint, as a separate determining factor, was not assessed.

Harty reported that the superficial peroneal nerve can be identified with plantar flexion and inversion. In one of the figure legends, it was stated that this nerve could be identified in about 20 % of the ankles. It is unclear how this number was obtained, and no references were given [14].

Since complication rates of anterior ankle arthroscopy are relatively high, ranging from 8 to 17 %, and mainly involved iatrogenic damage to the superficial peroneal nerve [2, 4, 6, 8, 10–13, 14, 16–18, 21, 27], different clinical tests were described to visualize this nerve during physical examination [3, 7, 11, 14, 17, 22, 24]. Preoperative identification of the superficial peroneal nerve could prevent iatrogenic damage during anterolateral portal placement in anterior ankle arthroscopy [23], external fixator applications, surgical decompressions for compartment syndromes, rapid localization prior to local anaesthetic blocks [22], and in open reduction and internal fixation of distal fibula fractures [19]. One should be aware, however, that marking the nerve in combined maximal plantar flexion and inversion can provide a false sense of safety. The position of this nerve changes with the position of the ankle. The superficial peroneal nerve moves on average 2 mm medially with maximal plantar flexion and inversion of the ankle, as compared to the neutral ankle position [9].

One of the study limitations is the single observer identification of the superficial peroneal nerve in the volunteers. The examiner was, however, trained in the local anatomy and performed dissections assisted by an experienced anatomist (PG) to avoid wrong identification. Also definitions were included in the study protocol to distinguish the superficial peroneal nerve from the surrounding anatomical structures such as the veins and tendons. The rate of inter- and intra-observer reliability remains to be determined.

Identification of the superficial peroneal nerve preoperatively may prevent iatrogenic damage while making the incisions for the arthroscopic portals [23]. The superficial peroneal nerve is more difficult to identify in patients with a higher BMI. This group of patients may, as a consequence, have an increased risk of iatrogenic superficial peroneal nerve damage. Currently no literature supports this hypothesis, necessitating further studies to elicit this.

## Conclusion

The best clinical test to identify the superficial peroneal nerve is forced ankle plantar flexion and inversion; thereby, the nerve can be identified in about six out of ten ankles by palpation. Increasing BMI negatively influences the identification of the superficial peroneal nerve by clinical examination.
